# Unusual Paraneoplastic Syndrome Accompanies Neuroendocrine Tumours of the Pancreas

**DOI:** 10.1155/2011/309149

**Published:** 2011-05-10

**Authors:** Helga Bertani, Alessandro Messerotti, Fabrizio Di Benedetto, Raffaele Manta, Milena Greco, Federica Casoni, Luisa Losi, Rita Conigliaro

**Affiliations:** ^1^Endoscopia Digestiva, Nuovo Ospedale Civile S. Agostino-Estense, Via Giardini 1355, 41100 Modena, Italy; ^2^Centro Trapianti di Fegato e Multiviscerale, Università di Modena e Reggio Emilia, Italy; ^3^Neurologia, Nuovo Ospedale Civile S. Agostino Estense, Modena, Italy; ^4^Anatomia Patologica, Università di Modena e Reggio Emilia, Italy

## Abstract

Neuroendocrine tumours comprise a small percentage of pancreatic neoplasia (10%) (1). Diagnosis of neuroendocrine tumours is difficult, especially if the tumours are small and nonfunctional. CT scans, MRI, and nuclear scans are sufficiently sensitive assessment tools for tumours with diameters of at least 2 cm; otherwise, the sensitivity and specificity of these techniques is less than 50% (2). Myasthenia gravis (MG) is a heterogeneous neuromuscular junction disorder that is primarily caused when antibodies form against the acetylcholine receptors (Ab-AchR). MG can develop in conjunction with neoplasia, making MG a paraneoplastic disease. In those cases, MG is most commonly associated with thymomas and less frequently associated with extrathymic malignancies. The mechanism underlying this paraneoplastic syndrome has been hypothesized to involve an autoimmune response against the tumour cells (3). No published reports have linked malignant pancreatic diseases with MG. Here, we report the case of a young woman, negative for Ab-AchR, with a neuroendocrine tumour in the pancreatic head, who experienced a complete resolution of her MG-like syndrome after surgical enucleation of the tumour.

## 1. Introduction

Advances in radiological imaging for the evaluation of patients suspected to have pancreatic tumours have led to increased detection of this type of tumour [1]. However, neuroendocrine tumours of the pancreas are usually very small and standard radiological techniques are not sufficiently sensitive [2]. If the neuroendocrine tumours are functional, their presentation is usually quite typical and the presenting symptoms can guide us to search for tumours in the small bowel, gastric wall, or pancreatic gland. When the symptoms are unusual or the tumour is nonfunctional, the diagnosis is much more challenging.

## 2. Case Report

A 32-year-old woman with a previous history of thyroidectomy for a multinodular goitre, within the past year, was under the supervision of a neurologist for weakness, symmetric eyelid ptosis, and a progressive loss of strength consistent with myasthenia gravis (MG). CT scan of the neck revealed a fatty infiltration of the thymus gland. The patient had a positive Tensilon test. The cardiac, thoracic, and abdominal examinations were unremarkable. 

The patient started a therapy of 60 mg pyridostigmine, four times daily (Mestinon), achieving a partial clinical response but worsening of the symptoms (eyelid ptosis) after 3 months. IgG therapy (25 g/day for 5 days) was initiated, and a partial clinical response was achieved. This unusual course, in conjunction with a negative test for Ab-AchR, led to additional investigations. 

**Figure 1:**
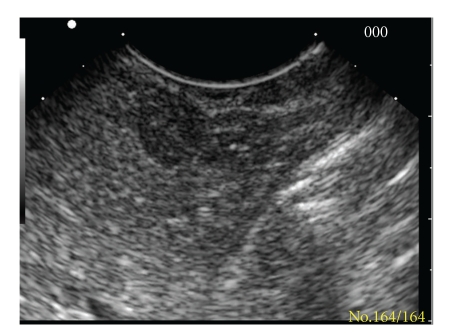
Endoscopic ultrasound of the hypoechoic lesion in the pancreatic head.

The patient underwent a PET scan that revealed a “captatio” in the head of the pancreas. She was then referred to our Endoscopy Unit for an endoscopic ultrasound (EUS). EUS revealed a small hypoechoic lesion in the head of the pancreas, 9.3 mm × 8 mm, with regular borders (Figure 1). The lesion was located near the pancreatic duct. The pancreatic body and tail were unremarkable. At the same time, a fine-needle aspiration biopsy (FNAB) of the lesion was performed with a 22 G needle, with 2 passes. After EUS, the patient underwent endoscopic retrograde cholangiopancreatography with sphincterotomy and pancreatic duct cannulation. We performed a pancreatic juice aspiration and conducted tests for serum carcinoembryonic antigen (CEA), chromogranin, and carbohydrate antigen (CA) 19.9. 

**Figure 2:**
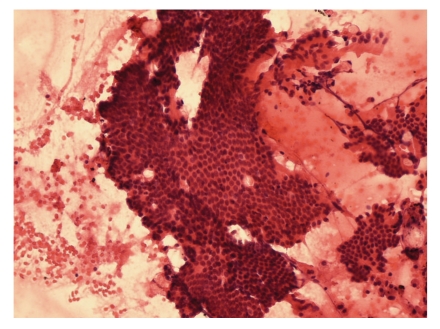
Hematoxylin-eosin staining of the specimen obtained with fine-needle aspiration. Magnification = 20x.

**Figure 3:**
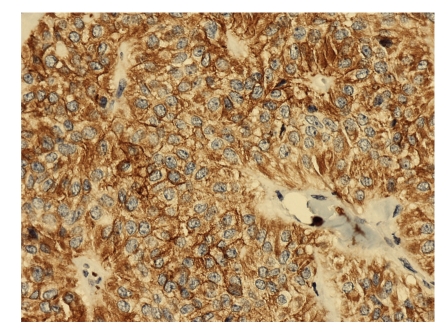
Chromogranin staining of the specimen obtained with fine-needle aspiration. Magnification = 20x.

**Figure 4:**
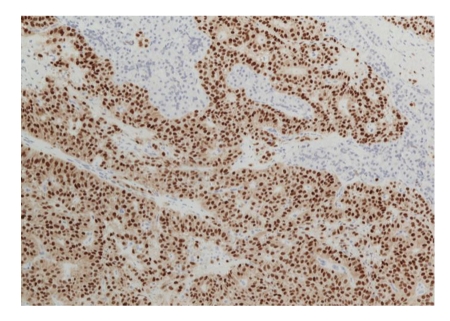
Progesterone staining of the surgical specimen.

Six hours after the procedure, the patient developed acute abdominal pain, with elevations in serum amylase and lipase. A CT scan revealed a dishomogeneous pancreatic parenchyma, which suggested edematous pancreatitis with abdominal, pleural, and pericardial effusion. Conservative treatment was performed with fast fluid resuscitation, gastric aspiration probe, and IV administration of piperacillin/tazobactam (4.5 g, twice). Chromogranin, CEA, Ca19.9, and neurone-specific enolase levels in the blood sample and pancreatic juice were within normal limits. Tests for 5-hydroxyindoleacetic acid and vanilmandelic acid in the urine were negative. The cytopathologist confirmed a well-differentiated neuroendocrine tumour in the sample obtained through FNAB (Figures 2, 3, and 4). The patient was discharged after 2 weeks, and the surgeons planned for enucleation of the small lesion in the pancreatic head. 

One week before surgery, we performed EUS with puncture of the lesion and insertion of Visicoil (IBA) to help localize the lesion during surgery. The patient underwent surgery with intraoperative ultrasound and successful localization of the lesion in the pancreatic head. The patient was discharged after 2 weeks, with a small pancreatic fistula that healed spontaneously in 2 weeks. Neurologist decided to discontinue Pyridostigmine 1 year after surgery, and the patient has been symptom-free for 3 years after-surgery.

## 3. Discussion

MG is an immune-mediated neurological disease. The most common symptoms at onset include eyelid ptosis, muscular weakness, and fatigability. The prevalence of the disease in the USA is reportedly 20 per 100,000 people in the general population, but the true prevalence is probably higher because MG frequently goes undiagnosed. Women are more often affected with MG in the second and third decades of life, whereas men are more often affected in the sixth decade. MG is frequently associated with positive tests for Ab-AChR but, in 15% of patients with generalized MG, Ab-AChR tests are negative [3]. Although removal of the primary tumour may be complete, the neurological symptoms usually do not disappear and recurrence of MG symptoms is quite frequent. The mechanism underlying this phenomenon involves an autoimmune response. MG is sometimes considered a paraneoplastic syndrome associated with cancers of the thymus and extrathymus [3]. An underlying neoplastic disease must be suspected if tests of Ab-AChR are negative and occur in conjunction with atypical onset of symptoms. 

Neuroendocrine tumours are considered as rare neoplasia arising from the gut. They are classified as functional or nonfunctional according to the presence or absence, respectively, of clinical symptoms associated with the production and secretion of various neuroendocrine mediators (serotonin, somatostatin, chromogranin, gastrin, insulin, glucagon, histamine, glucose-dependent insulinotropic peptide, neurotensin, vasoactive intestinal peptide, pancreatic polypeptide). Only 10% of neuroendocrine tumours are functional, and the symptoms described most frequently for neuroendocrine syndromes include flushing (80%), diarrhea (75%), weight loss, nettle rash, itching, sweating, hypercalcaemia, acromegaly, or Cushing syndrome, which are due to the production of specific neuromediators. 

Diagnosis of neuroendocrine tumours is difficult, especially if the tumour is small and nonfunctional. Published reports suggest that CT scans have a sensitivity range of 11–66% and a specificity range of 83–100% whereas MRI has a sensitivity range of 20–25% and a specificity range of 89–100%. Nuclear scans have good sensitivity (58–100%) if the tumour diameter is at least 2 cm; otherwise, the sensitivity is less than 50%. A specificity range has not been reported for nuclear scans. EUS can be used to diagnose very small tumours, and published reports of prospective and retrospective studies suggest that this technique has a sensitivity range of 57–100%. EUS is extremely useful for identifying pancreatic head tumours [4–6]. However, the sensitivity decreases from 92% for tumours in the pancreatic head to 40% for tumours in the pancreatic tail [7]. During EUS, it is possible to perform FNAB to obtain tissue specimens for subsequent cytopathological and immunohistochemical analyses [8]. The accuracy of EUS-FNAB for the diagnosis of pancreatic carcinoma and neuroendocrine tumours is reported to be 80%–95% [9–12] and 46%–83%, respectively [13, 14]. 

There are no published reports regarding neurological disorders associated with pancreatic neoplasms, only reports regarding pancreatic cancer in association with a paraneoplastic syndrome (deep vein thrombosis, coagulation disorders, hypercalcaemia, acromegaly, or Cushing syndrome). This report is the first description of a small neuroendocrine tumour of the pancreas, diagnosed via EUS, that was associated with paraneoplastic MG. However, two points must be considered regarding the clinical relevance of this case report. First, this is a rare form of pancreatic tumour, presenting with a better prognostic score than pancreatic adenocarcinoma. The better prognosis was due to the relatively small diameter of the tumour at the time of diagnosis, and to a well-differentiated type of this tumour with low mythotic index. Many reports of symptoms attributed to the secretion of neuromediators have been based on histopathological examination of the neuroendocrine tumour, but a neurological syndrome has never been reported. A retrospective analysis of the data from patients that underwent surgical resection of neuroendocrine neoplasia, registered as “asymptomatic,” may reveal neurological symptoms missed during the previous analysis. Second, a complete response to surgical therapy is quite uncommon with neurological disorders. Paraneoplastic neurological syndrome is quite common in patients with breast cancer, but these patients rarely recover after surgical ablation of the primary tumour, even after many years of disease-free survival. In the present case, pharmacological therapy was discontinued after 1 year, and the patient is still symptom-free after 3 years. The differences in outcome are probably due to differences in the underlying pathogenetic pathways; more studies are needed to fully understand these syndromes.
